# Dupilumab improves clinical and histologic features of eosinophilic esophagitis prior to 12 weeks of treatment

**DOI:** 10.1002/clt2.12333

**Published:** 2024-01-17

**Authors:** Twan Sia, Amanda Miller, Leeon Bacchus, Jennie Young, Aditya P. Narayan, Rachel Solecki, Jerry Fu, Yuting Jiang, Raisa Khuda, Stanley Liu, Kathleen Love, Shibani Mallik, Amina Sara Matmatte, Paige McDonald, Tanvi Telukunta, Alyssa Roby, Saad Shami, Michelle Zheng, Madison Headen, John Leung

**Affiliations:** ^1^ Stanford University School of Medicine Stanford California USA; ^2^ Boston Specialists Boston Massachusetts USA

To the editor

Dupilumab is a human monoclonal antibody against interleukin‐4 receptor alpha subunit. Dupilumab is an approved treatment for inducing remission of eosinophilic esophagitis (EoE).^1^ EoE histologic remission with dupilumab has only been demonstrated in patients after at least 12 weeks of treatment.^2^, ^3^, ^4^, ^5^, ^6^ Current guidelines recommend waiting for histologic re‐evaluation of EoE until after 20–24 weeks of dupilumab.^1^ It is unknown if increasing dupilumab treatment length improves its efficacy. Because histologic re‐evaluation of EoE requires invasive biopsies, and inducing remission of EoE is important to prevent progressive esophageal damage, research investigating the effects of dupilumab on EoE prior to 12 weeks of treatment is warranted.

We conducted a retrospective study at a single medical clinic. The electronic medical record was searched between 2017 and 2023 using International Classifications of Disease, 10th revision code K20.0 eosinophilic esophagitis. We excluded patients who had (1) never started dupilumab; (2) no histologic confirmation of EoE defined by ≥ 15 eos/hpf; or (3) no histologic re‐evaluation of EoE while on dupilumab. Histologic evaluation of EoE assessed at least 2 biopsies each of the proximal, middle, and distal esophagus. Endpoints were peak eosinophil counts (eosinophils per high‐power field; eos/hpf), EoE endoscopic reference scores (EREFS), and a composite symptom score in which each symptom (dysphagia, food impaction/choking, regurgitation/vomiting, heartburn/chest pain, and abdominal pain) was graded (0 = absent, 1 = mild, 2 = moderate, and 3 = severe) and summed. This study was deemed exempt from institutional review board approval by the WCG IRB.

From the electronic medical record, 658 patients with EoE were identified, of which 534 had never initiated dupilumab, 6 did not have histologic confirmation of EoE, and 39 did not have a repeat histologic evaluation after dupilumab initiation. Therefore, 79 patients were included in this study. The median age was 27.6 years (Q1 to Q3, 21.8–36.1), 48 patients (60.8%) were male, and 12 patients (15.2%) were pediatric (Table 1). Sixty patients (75.9%) had an atopic comorbidity, including allergic rhinitis (43 patients, 54.4%), asthma (27 patients, 34.2%), atopic dermatitis (13 patients, 16.5%), and food allergies (30 patients, 38.0%).

**TABLE 1:** Changes in composite symptom scores, peak eosinophil counts, and EoE endoscopic reference scores in all included patients and stratified by those on dupilumab for 0–12, 12–24, and greater than 24 weeks.

Characteristics, median (Q1 to Q3)	All included patients (*n* = 79)	Patients on dupilumab for 0–12 weeks (*n* = 12)	Patients on dupilumab for 12–24 weeks (*n* = 38)	Patients on dupilumab for longer than 24 weeks (*n* = 29)	*p*‐value
Change in composite symptoms scores	−5.0 (−6.0 to −3.0)	−5.5 (−6.0 to −4.0)	−5.0 (−6.0 to −3.8)	−3.0 (−6.0 to −2.0)	0.1350
Changes in peak eosinophil count (eos/hpf)	−33.0 (−54.0 to −18.0)	−24.5 (−54.3 to −5.5)	−35.0 (−49.3 to −24.0)	−45.0 (−78.0 to −25.0)	0.0746
Changes in EoE endoscopic reference scores	−1.0 (−4.0 to 0.0)	−2.0 (−4.0 to 0.50)	−1.0 (−3.0 to 0.0)	−1.0 (−2.0 to −0.25)	0.8771

*Note*: Change in EoE endoscopic reference scores is only available for 15 patients. *p* value corresponds to Kruskal‐Wallis test comparing patients on dupilumab for 0–12, 12–24, and greater than 24 weeks.

Patients were on dupilumab for median 22.7 weeks (Q1 to Q3, 16–26.7). Dosages included 300 mg every week (71 patients, 89.9%), 300 mg every other week with a loading dose of 600 mg for atopic dermatitis (7 patients, 8.9%), and 200 mg every other week with a loading dose of 400 mg for atopic dermatitis (1 patient, 1.3%).

Of 79 patients, 12 patients (15.2%) were on dupilumab for 0–12 weeks. Patients on dupilumab for 0–12 weeks had a median composite symptom score of 5.5 (Q1 to Q3, 4–6), which significantly decreased to 0 (Q1 to Q3, 0–1; Wilcoxon matched‐pairs signed rank test, *p* = 0.000488) on dupilumab. Median peak eosinophil counts in patients on dupilumab for 0–12 weeks significantly decreased from 44.5 eos/hpf (Q1 to Q3, 32.5–53.5) at baseline to 2 eos/hpf (Q1 to Q3, 0–15.5; Wilcoxon matched‐pairs signed rank test, *p* = 0.000977) on dupilumab. Endoscopic reference scores were only available for 15 patients (19%) in our cohort. In patients on dupilumab for 0–12 weeks, EREFS did not significantly decrease from baseline (median, 2; Q1 to Q3, 1–4) versus on dupilumab (median, 0; Q1 to Q3, 0–1.5; Wilcoxon matched‐pairs signed rank test, *p* = 0.25). However, change in EREFS was also insignificant in patients on dupilumab for 12–24 weeks (*p* = 0.13), and greater than 24 weeks (*p* = 0.25), suggesting insignificance may be due to low *n*. Therefore, dupilumab may induce histologic remission and clinical benefit in patients prior to 12 weeks of treatment.

There were no significant differences in changes in median composite symptom score (*p* = 0.1350), peak eosinophil count (*p* = 0.0746); and EREFS (*p* = 0.8771) between patients on dupilumab between 0 and 12, 12–24, and greater than 24 weeks (Table 1). In terms of histologic response, 9 patients (75%) were histologically responsive in the 0–12 weeks group, 28 patients (73.7%) were responsive in the 12–24 weeks group, and 26 patients (89.7%) were responsive in the longer than 24 weeks group. There was no significant difference in the proportion of histologic response between the 3 groups (Fisher's exact test, *p* = 0.2569).

Subanalysis in 7 patients with >1 histologic evaluations on dupilumab is summarized in Figure 1. Three patients who were histologically unresponsive to dupilumab at early timepoints (Patient 4 between 0 and 12 weeks, Patient 3 and 5 between 12 and 24 weeks) were responsive after 24 weeks of treatment without addition of combination therapy. In contrast, Patient 1 was unresponsive at 0–12 weeks of dupilumab and remained unresponsive after over 24 weeks of dupilumab. Patient 2 and 6 had started combination therapy with omeprazole or mometasone, respectively. Therefore, their histologic remission may be due to combination therapy. Our subanalysis suggests that certain patients who are histologically unresponsive at early EGDs may or may not respond at later timepoints. Further research is needed to predict which patients benefit from repeat EGDs.

**FIGURE 1:**
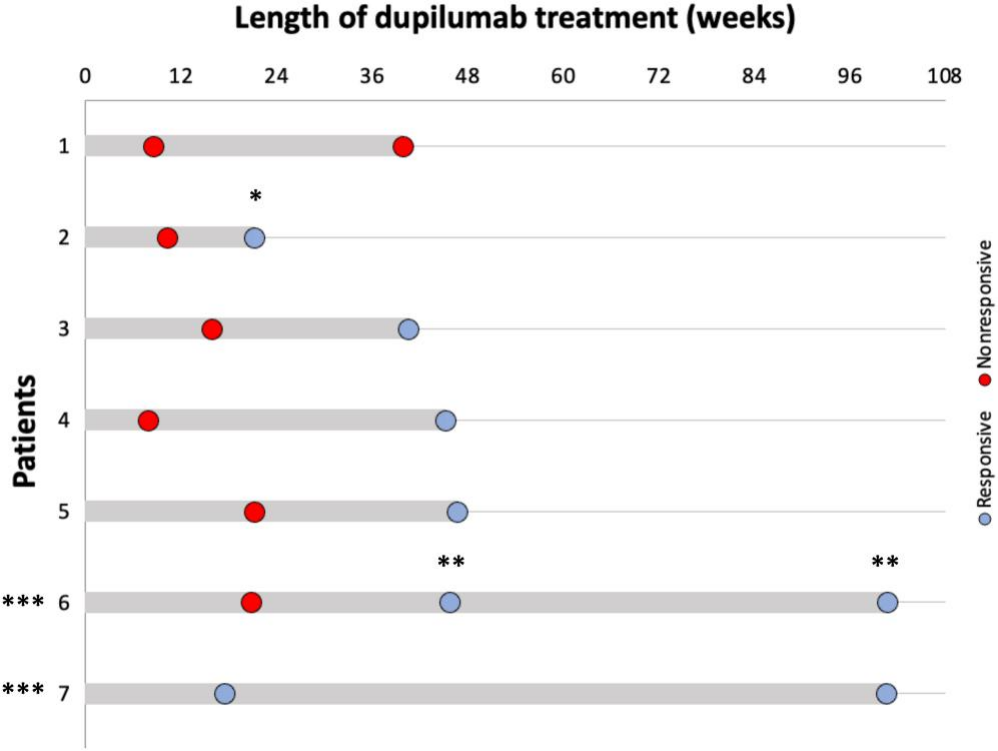
Swimmer plot of patients with eosinophilic esophagitis that had multiple histologic evaluations while on dupilumab therapy. * indicates combination therapy with mometasone 1.6 mg twice daily. ** indicates combination therapy with omeprazole 20 mg once daily. *** indicates dupilumab 300 mg once every 2 weeks, loading dose 600 mg, as opposed to dupilumab 300 mg once a week.

In conclusion, dupilumab induced histologic remission and clinical benefit before 12 weeks of treatment, and there were no significant differences in clinical, histologic, or endoscopic changes between patients on dupilumab for 0–12 weeks, 2–24 weeks, and greater than 24 weeks. It may be beneficial to identify treatment response earlier than previous guidelines indicate.^1^ Further research should investigate the appropriate window of treatment before repeat EGDs are performed.

## AUTHOR CONTRIBUTIONS

**Twan Sia**: Conceptualization (equal); data curation (equal); formal analysis (equal); investigation (equal); methodology (equal); validation (equal); visualization (equal); writing—original draft (equal); writing—review and editing (equal). **Amanda Miller**: Data curation (equal); formal analysis (equal); validation (equal); visualization (equal); writing—original draft (equal); writing—review and editing (equal). **Leeon Bacchus**: Data curation (equal); formal analysis (equal); validation (equal); visualization (equal); writing—original draft (equal); writing—review and editing (equal). **Jennie Young**: Data curation (equal); validation (equal); visualization (equal); writing—review and editing (equal). **Aditya P. Narayan**: Data curation (equal); validation (equal); visualization (equal); writing—review and editing (equal). **Rachel Solecki**: Investigation (equal); validation (equal); writing—review and editing (equal). **Jerry Fu**: Investigation (equal); validation (equal); writing—review and editing (equal). **Yuting Jiang**: Investigation (equal); validation (equal); writing—review and editing (equal). **Raisa Khuda**: Investigation (equal); validation (equal); writing—review and editing (equal). **Stanley Liu**: Investigation (equal); validation (equal); writing—review and editing (equal). **Kathleen Love**: Investigation (equal); validation (equal); writing—review and editing (equal). **Shibani Mallik**: Investigation (equal); validation (equal); writing—review and editing (equal). **Amina Sara Matmatte**: Investigation (equal); validation (equal); writing—review and editing (equal). **Paige McDonald**: Investigation (equal); validation (equal); writing—review and editing (equal). **Tanvi Telukunta**: Investigation (equal); validation (equal); writing—review and editing (equal). **Alyssa Roby**: Investigation (equal); validation (equal); writing—review and editing (equal). **Saad Shami**: Investigation (equal); validation (equal); writing—review and editing (equal). **Michelle Zheng**: Investigation (equal); validation (equal); writing—review and editing (equal). **Madison Headen**: Investigation (equal); validation (equal); writing—review and editing (equal). **John Leung**: Conceptualization (equal); methodology (equal); project administration (equal); resources (equal); supervision (equal); writing—review and editing (equal).

## CONFLICT OF INTEREST STATEMENT

John Leung: is a consultant for Devine; Millimet and Branch Professional Education; Sanofi; Huron Consulting Services LLC; Takeda; Ribon Therapeutics; Tegus; Slingshot; Guidepoint; Cowen; AstraZeneca; Regeneron; and AbbVie. None of the other authors have relevant conflicts of interests to disclose.

## FUNDING INFORMATION

This research received no specific grant from any funding agency in the public, commercial, or not‐for‐profit sectors.

## Data Availability

All relevant de‐identified data and study materials are stored in a HIPPA‐compliant, password‐protected, cloud‐based storage. Access to these files will be provided upon reasonable request to the corresponding author, John Leung.
